# Dr. Andrew Kamerling

**Published:** 1918-02

**Authors:** 


**Dr. Andrew Kamerling, Buffalo** 1866, died at his home in Buffalo, Dec. 6, aged 78.

